# Molecular and Cellular Biomarkers of COVID-19 Prognosis: Protocol for the Prospective Cohort TARGET Study

**DOI:** 10.2196/24211

**Published:** 2021-03-04

**Authors:** Patricia Kurizky, Otávio T Nóbrega, Alexandre Anderson De Sousa Munhoz Soares, Rodrigo Barbosa Aires, Cleandro Pires De Albuquerque, André Moraes Nicola, Patrícia Albuquerque, Andréa Teixeira-Carvalho, Luciana Ansaneli Naves, Wagner Fontes, Isabelle Souza Luz, Liza Felicori, Ana Paulo Monteiro Gomides, Dayde Lane Mendonça-Silva, Laila Salmen Espindola, Olindo Assis Martins-Filho, Sheila Maria Barbosa de Lima, Licia Maria Henrique Mota, Ciro Martins Gomes

**Affiliations:** 1 Programa de Pós-graduação em Ciências Médicas da Faculdade de Medicina University of Brasília Brasilia Brazil; 2 Hospital Universitário de Brasília University of Brasília Brasília Brazil; 3 Faculdade de Ceilândia University of Brasília Brasília Brazil; 4 Fundação Oswaldo Cruz FIOCRUZ-Minas Belo Horizonte Brazil; 5 Instituto de Ciências Biológicas University of Brasília Brasília Brazil; 6 Instituto de Ciências Biológicas Federal University of Minas Gerais Belo Horizonte Brazil; 7 Centro Universitário de Brasília Brasília Brazil; 8 Faculdade de Ciências da Saúde University of Brasília Brasília Brazil; 9 Instituto de Tecnologia em Imunobiológicos Bio-Manguinhos FIOCRUZ Rio de Janeiro Brazil

**Keywords:** COVID-19, TARGET, cytokine profile, neutrophil function, thromboelastometry, neutralizing antibodies, metabolomics, proteomics, biomarker, prognosis, design, cohort, virus, immunology, immune system, genetics

## Abstract

**Background:**

Since the beginning of the COVID-19 pandemic, the world’s attention has been focused on better understanding the relation between the human host and the SARS-CoV-2 virus, as its action has led to hundreds of thousands of deaths.

**Objective:**

In this context, we decided to study certain consequences of the abundant cytokine release over the innate and adaptive immune systems, inflammation, and hemostasis, comparing mild and severe forms of COVID-19.

**Methods:**

To accomplish these aims, we will analyze demographic characteristics, biochemical tests, immune biomarkers, leukocyte phenotyping, immunoglobulin profile, hormonal release (cortisol and prolactin), gene expression, thromboelastometry, neutralizing antibodies, metabolic profile, and neutrophil function (reactive oxygen species production, neutrophil extracellular trap production, phagocytosis, migration, gene expression, and proteomics). A total of 200 reverse transcription polymerase chain reaction–confirmed patients will be enrolled and divided into two groups: mild/moderate or severe/critical forms of COVID-19. Blood samples will be collected at different times: at inclusion and after 9 and 18 days, with an additional 3-day sample for severe patients. We believe that this information will provide more knowledge for future studies that will provide more robust and useful clinical information that may allow for better decisions at the front lines of health care.

**Results:**

The recruitment began in June 2020 and is still in progress. It is expected to continue until February 2021. Data analysis is scheduled to start after all data have been collected. The coagulation study branch is complete and is already in the analysis phase.

**Conclusions:**

This study is original in terms of the different parameters analyzed in the same sample of patients with COVID-19. The project, which is currently in the data collection phase, was approved by the Brazilian Committee of Ethics in Human Research (CAAE 30846920.7.0000.0008).

**Trial Registration:**

Brazilian Registry of Clinical Trials RBR-62zdkk; https://ensaiosclinicos.gov.br/rg/RBR-62zdkk

**International Registered Report Identifier (IRRID):**

DERR1-10.2196/24211

## Introduction

COVID-19 emerged in the city of Wuhan, Hubei, China and spread worldwide over the next several months [1]. Originally reported by the World Health Organization (WHO) as a pneumonia outbreak of undetermined origin, COVID-19 had its epidemiological status revised by the WHO as a Public Health Emergency of International Concern by the end of January 2020 and as a pandemic at the beginning of March 2020 [2]. The etiologic agent was identified as a new coronavirus of the *Betacoronavirus* genus, named SARS-CoV-2, due to its structural similarity to severe acute respiratory syndrome–related coronavirus (SARS-CoV), which also accounted for an outbreak in China in 2002-2003 [3]. The disease is characterized by fever, cough, and dyspnea, and can progress to pulmonary failure and lead to liver, heart, and kidney damage. Clinical management of severe cases requires respiratory assistance [4].

Angiotensin converting enzyme-2 (ACE2) mediates SARS-CoV-2 (and other coronaviruses) entry via clathrin-mediated endocytosis into type-2 pneumocytes and macrophages of the lung milieu [5,6]. ACE2 is a membrane-bound enzyme that converts active angiotensin II to inactive angiotensin (1-7). Thus, ACE2 blocks the unfavorable effects of angiotensin II action, including classic vasoconstriction in addition to inflammation and thrombosis. SARS-CoV-2 binding markedly impairs ACE2 catalytic activity by competitive inhibition, with increased in situ pulmonary inflammation and coagulation as detrimental outcomes of weakened counter regulation of the angiotensin II/AT1 receptor axis [7].

Hence, COVID-19 resembles a hyperimmune syndrome defined by potentially lethal hypercytokinemia due to overproduction of a set of proinflammatory mediators, including interleukins (IL-1β, IL-2, IL7), granulocyte colony stimulating factor, interferon-gamma (IFN-γ), monocyte chemoattractant protein-1 (MCP-1), and tumor necrosis factor-alpha (TNF-α), which culminates in multi-organ failure [8,9]. In addition, the Th17-type response plays an important role in severe cases due to an additional proinflammatory overflow resulting from supraphysiological IL-1β, IL-6, and TNF-α levels that contribute to edema formation [10]. The role of cortisol and prolactin in the modulation of the immune system has long been described [11,12], but their participation (if any) in the COVID-19–related “cytokine storm” remains unknown. Moreover, given the intimate interplay of inflammation with blood clotting, severe patients usually show a hypercoagulable profile that leads to an increased frequency of pulmonary thromboembolism, deep vein thrombosis, and even heart and brain ischemia events [13,14].

In this context, it is also no surprise that the first treatment that reduced COVID-19 mortality, in a large randomized controlled trial (RECOVERY [Randomised Evaluation of COVID-19 Therapy]) [12,15], was dexamethasone, a corticosteroid [12]. However, it also showed that the effect seems to depend on the clinical stage of the disease, highlighting the need for further investigations into the course of the inflammatory response. Other anti-inflammatory treatments such as tocilizumab, an IL-6 receptor-blocking humanized antibody used to treat rheumatoid arthritis, were used (out of compassion) in patients with severe COVID-19 in China and Italy [16-18], with some clinical improvement. Similarly, another drug under evaluation is baricitinib, a JAK2 inhibitor approved for myeloproliferative neoplasms and rheumatoid arthritis that reduces Th17-type cytokine secretion by blocking AP2-associated protein kinase 1 [19]. However, the clinical efficacy of baricitinib against COVID-19 has yet to be proven. These studies support the assumption that a cytokine “storm” not only plays a role in COVID-19 pathophysiology but also has predictive prognostic value to monitor the evolution of mild and severe cases.

New metabolomics protocols can characterize metabolic pathways and provide a broad view of the impact of SARS-CoV-2 on the body. Both metabolome and proteome composition are dynamic and reflect genome expression under specific conditions [20-22]. Furthermore, these innovative combination protocols can help in understanding the metabolic profile of a given patient or group of patients [23] from urine, saliva, and blood samples [24]. The chemical profile substantially contributed to biological and medical research, leading to advancements in clinical medical practice [23].

This paper describes the design and rationale of a multicenter prospective cohort study aimed at evaluating the molecular and cellular immune signature of COVID-19 by assessing serum inflammatory mediators and regulatory chemokines of Brazilian patients with different clinical forms of the disease.

## Methods

### Study Design and Population

TARGET is registered in the Brazilian Clinical Trials database with registry code RBR-62zdkk and constitutes a multicenter, prospective cohort study of consecutive COVID-19 cases, consisting of participants recruited from secondary and tertiary health care facilities in Brazil, namely, the University Hospital of Brasília (University of Brasília [UnB]) and the public regional hospitals of Asa Norte, Gama, Santa Maria, and Taguatinga (Brasília, Distrito Federal). Clinical procedures will be conducted according to a standard protocol approved by local institutional review boards under accession number CAAE 30846920.7.0000.0008, with participants included after voluntary signing of an informed consent form. The study protocol follows the recommendations of the STROBE (Strengthening the Reporting of Observational Studies in Epidemiology) statement for observational studies [16].

### Recruitment

**Figure 1:**
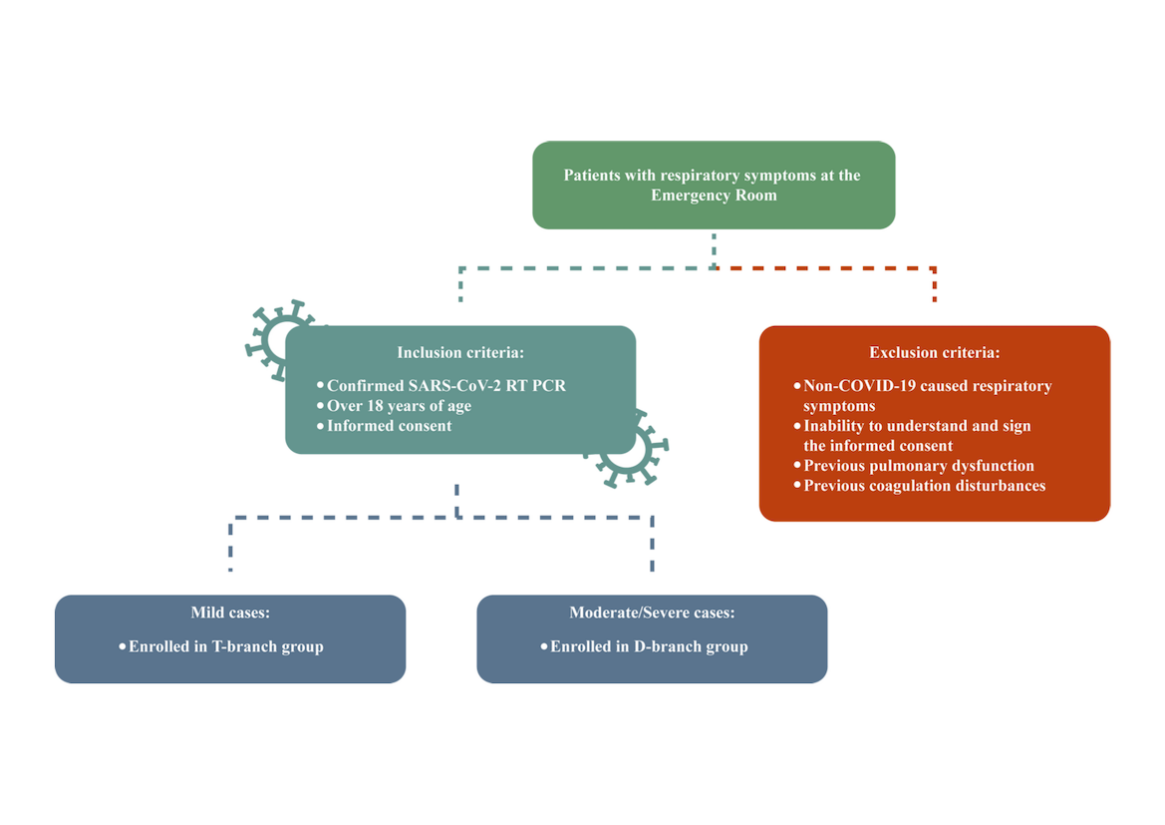
Inclusion and exclusion criteria flowchart. The T-branch group is patients referred to the emergency room with mild or moderate forms of COVID-19 not requiring hospitalization. The D-branch group is patients referred to the emergency room with severe or critical forms of COVID-19 requiring hospitalization.

Inclusion criteria will include the following: laboratory confirmation of COVID-19 by reverse transcription polymerase chain reaction (RT-PCR; KIT Molecular SARS-CoV2 [E/RP]; Bio-Manguinhos), a signed informed consent form, and older than 18 years. The exclusion criteria are as follows: patients with inconclusive, unavailable, or solely serological laboratory diagnosis of COVID-19; a dementia condition (Alzheimer, Parkinson, vascular frontotemporal, Lewy body, or others); schizophrenia or schizoaffective disorders with psychotic characteristics; and patients with previously known congenital hemorrhagic diseases or thrombophilia or use of anticoagulants (for coagulation tests; Figure 1).

### Clinical Assessment Protocol

The clinical evaluation aims to identify the signs and symptoms associated with COVID-19 and monitor the status (mild/moderate or severe/critical) of the disease. Clinical assessments include identification of comorbidities, COVID-19–associated symptoms and treatment, use of medications in general, clinical chemistry findings, and life support regimen care. Severe cases are defined for the purposes of the study by dyspnea (respiratory rate >30 breaths per minute) coupled with any of the following criteria: pulsed wave oxygen saturation <93% at rest, PaO_2_/FiO_2_ ≤300 mmHg, respiratory failure requiring mechanical ventilation, multiple organ failure, shock, or admission to the intensive care unit. Patients who do not meet the criteria for severe/critical forms will be considered mild/moderate cases. No asymptomatic patients will be included (Textbox 1 and Table 1).

**Components being assessed for characterization of the following domains: symptoms, comorbidities, treatments, biochemical profile, immune biomarkers, and leukocyte phenotype and functionality in the TARGET protocol.**

**Symptoms**

Fever, chills, sneezing, sore throat, headache, cough, coryza, anosmia, dysgeusia, diarrhea, asthenia, nausea, vomit, dizziness, and others

**Comorbidities**

Pulmonary chronic disease, chronic cardiopathy, hypertension, diabetes, chronic kidney disease, pregnancy, neoplasms, smoking, alcohol ingestion, psoriasis, immunodepression, HIV, Dengue, hanseniasis, Zika virus, chikungunya, Chagas disease, yellow fever, leishmaniasis, malaria, H1N1, and others

**Previous medications**

Angiotensin converting enzyme inhibitors, angiotensin II receptor antagonists, nonsteroidal anti-inflammatory drugs, and others

**Treatments aimed at COVID-19**

Clinical support, dexamethasone, enoxaparin, chloroquine, antibiotics, ivermectin, nitazoxanide, lopinavir, ritonavir, remdesivir, immunoglobulin, plasmapheresis, anti-IL6, JAK inhibitor, and others

**Biochemical tests**

Leukocyte, lymphocyte and platelet counts, ferritin, lactate dehydrogenase, troponin, creatine kinase, aspartate aminotransferase, alanine aminotransferase, and creatinine

**Hormone evaluation**

Prolactin and cortisol

**Thorax computed tomography**

Ground-glass opacity (<25%, 25-50%, >50%)

Consolidation

**Serum immune biomarkers**

Chemokines: CXCL8, CCL11, CCL3, CCL4, CCL2, CCL5, and CXCL10; inflammatory cytokines: IL-1β, IL-6, TNF-α, IL-12p70, IFN-gamma, IL-17A, and IL-15; regulatory cytokines: IL-1Ra, IL-4, IL-5, IL-9, IL-10, and IL-13; and cell growth factors: IL-2, IL-7, FGF-basic, PDGF, VEGF, G-CSF, and GM-CSF

**Gene expression assays**

IFN-γ, IL-12, IL-17a, TNF-α, IL-6, IL-4, IL-5, IL-10, IL-1b, RANTES, MCP-1, MIP, MIG, IP-10, and IL-8

**Leukocyte phenotyping**

CD3, CD4, CD8, HLA-DR, CD25, CD19, CD5, CD27, CD38, PD-1, CD28, CD14, CD16, and CD56

**Leukocyte functionality**

IL-1β, IL-6, TNF-α, IFN-α, IL-5-PE, and IL-10

**Metabolomic analysis**

Metabolic profile/pathways

**Neutrophils**

Isolation, reactive oxygen species production, phagocytosis, neuroendocrine tumors production, migration assay, neutrophil proteomics

**Table 1:** The TARGET protocol will collect information about a series of characteristics and tests along the clinical evolution of patients with COVID-19. At each time point, the following data will be assessed.

Data registered	Day 0	Day 3	Day 9	Day 18
Age, gender, weight, and height	✓^a^	—^b^	—	—
Symptoms	✓	✓	✓	✓
Contact with SARS-CoV-2 patient	✓	—	—	—
Reverse transcription polymerase chain reaction	✓	—	—	—
Time of symptoms	✓	—	—	—
Comorbidities	✓	—	—	—
Medications in use	✓	✓	✓	✓
SpO2	✓	✓	✓	✓
Thorax computed tomography	✓	✓	✓	✓
Biochemical tests	✓	✓	✓	✓
Supplementary oxygen	✓	✓	✓	✓
Mechanical ventilation	✓	✓	✓	✓
Death	✓	✓	✓	✓
COVID-19 treatment	✓	✓	✓	✓
Immune biomarkers	✓	✓	✓	—
Leukocyte phenotyping	✓	✓	✓	—
Cortisol/prolactin levels	✓	✓	✓	✓
Immunoglobulin profile	—	✓	—	—
D-dimer	—	—	✓	—
Thromboelastometry	—	—	✓	—
Neutralizing antibodies	—	—	—	✓
Metabolomic analysis	✓	✓	✓	✓
Gene expression	—	✓	✓	—
Neutrophils	—	—	✓	—

^a^Indicates data was registered at this time point.

^b^Indicates data was not registered at this time point.

### Timeline

The TARGET protocol comprises two arms, referred to as the timeline (T)-branch and the days of hospitalization (D)-branch. For the purposes of this study, the T-branch refers to patients with mild/moderate forms that did not require hospitalization. The D-branch refers to patients with severe/critical forms who required hospital admission. Patients on the T-branch will be analyzed on days 0, 9, and 18 (T0, T9, and T18), while patients on the D-branch will be analyzed on days 0, 3, 9, and 18 (D0, D3, D9, and D18; Figure 1).

### Biological Samples

**Figure 2:**
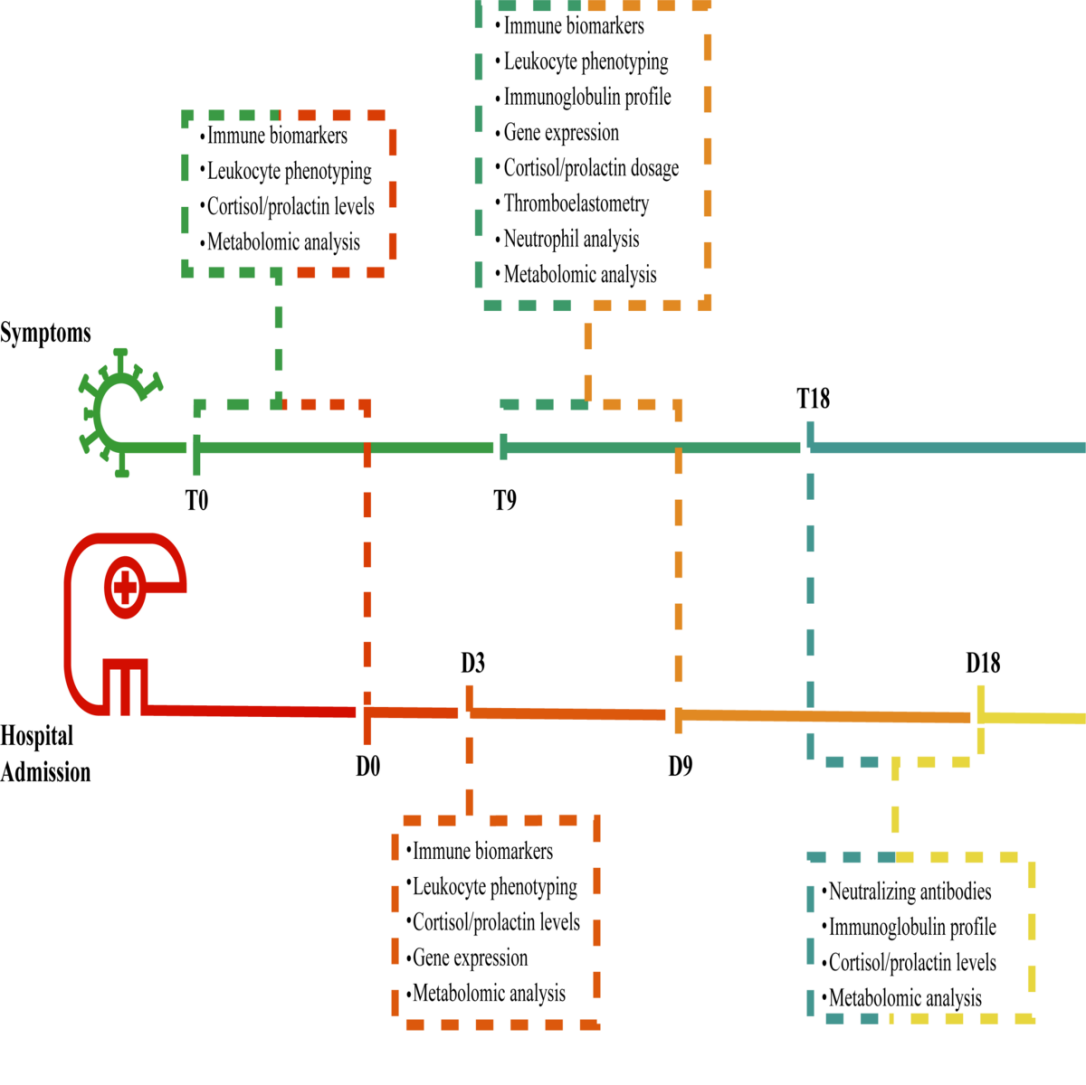
Timeline showing the times of blood tests and the analysis performed. The upper line represents the duration of symptoms, a parameter used in the group of patients enrolled for prospective follow-up, composed primarily of patients who will evolve with mild conditions. The lower line represents the blood test dates in the hospitalized group: patients who have already entered the study with moderate to severe conditions, usually from the fifth day of symptom onset.

The TARGET protocol encompasses the collection of blood samples in four different types of containers. For these purposes, whole peripheral blood samples will be collected in vacuum tubes without an anticoagulant (10 mL) to obtain serum samples for metabolomic and biochemical analysis, in sodium citrate (5 mL) for coagulation assays, and in heparin (10 mL) to obtain plasma samples and to isolate polymorphonuclear and peripheral blood mononuclear cells (PBMC). Samples will be collected at distinct time points as follows: day 0, at inclusion by confirmation of COVID-19; day 3, within 2-4 days after inclusion; day 9, within 7-10 days after inclusion; and day 18, within 15-20 days after inclusion (Figure 2).

### Preanalytical Sample Handling and Preprocessing

The whole blood samples will be processed to obtain serum or plasma samples and PBMC batches. Tubes will be centrifuged at 1900 g for 10 minutes at 22 °C to obtain serum or heparinized plasma and processed immediately within-facility for routine PBMC isolation and cryopreservation [17].

### Clinical Biochemistry and Hematologic Analysis

Plasma samples obtained in ethylenediamine tetraacetic acid will be immediately analyzed at local clinical laboratory facilities. Biochemical analysis will be performed with a Rocas Cobas E411 system using compatible reagents to assess the levels of ferritin, troponin T, creatine kinase, creatinine, aspartate aminotransferase, and alanine aminotransferase. All procedures will be performed according to the manufacturer’s recommendations. Cortisol and prolactin in serum samples will be quantified at the Sabin Medicina Diagnóstica (Brasília, Brazil) using the chemiluminescent method (Siemens, Advia Centaur). Hematological analyses will be performed using whole blood obtained in sodium citrate tubes in an ABBOTT Cell Dyn 3700 analyzer or equivalent with samples from days 0, 9, and 18.

**Figure 3:**
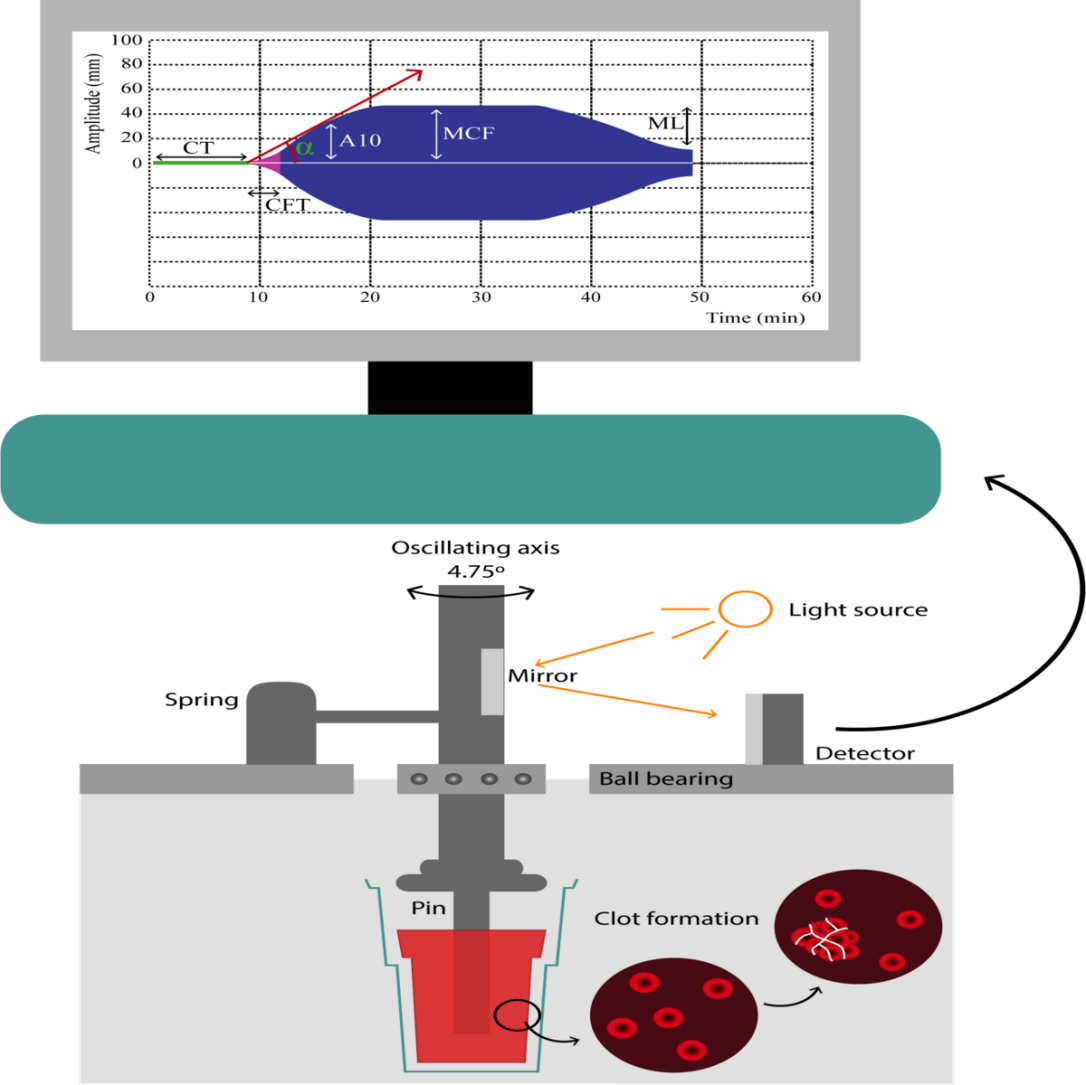
Thromboelastometry method for clot evaluation. A pin that spins around its own axis is put in contact with a citrated blood sample inside a cuvette. After recalcification and addition of a specific activator (depending upon the test), the clotting starts, and as it gets firmer, the spinning capacity of the axis is reduced, which is transformed by the system in a graphic representation of the clot, with increasing amplitude. As the fibrinolysis starts, the clot becomes less firm, which is represented as decreasing amplitude on the monitor. The extrinsic system assay (EXTEM)'s activator is thromboplastin and evaluates the extrinsic activation of coagulation. The intrinsic system assay's activator is elagic acid and evaluates the intrinsic activation of coagulation. The fibrinogen assay's activator is the same as EXTEM plus cytochalasin D, which inhibits platelet activity. This depicts only the participation of fibrinogen in the clot. The nonactivated coagulation assay is recalcified blood and is a nonactivated evaluation of coagulation. Circulating tissue factors, such as those expressed on monocytes in inflammatory states, will start the coagulation process. CT is the time frame from activation until an amplitude of 2 mm and represents thrombin formation. CFT represents the dynamic formation of thrombin and is the time frame between 2 mm and 20 mm of clot amplitude. A10 represents the clot amplitude 10 minutes after initiation and is directly related to MCF, enabling the clinic to make important decisions. MCF is the maximum amplitude of the clot and represents its main constituents, namely, fibrinogen and platelets. ML represents the percentage of clot reduction after initiation of fibrinolysis. Therefore, 60 minutes after initiation, thromboelastometry depicts important information about every phase of the coagulation process. A10: amplitude in 10 minutes; CFT: coagulation formation time; CT: coagulation time; MCF: maximum clot firmness; ML: maximum lysis.

The coagulation profile will be assessed at the DASA Laboratory (Brasília, Brazil) by thromboelastometry tests carried out within a 3-hour interval after blood collection [13,25] as follows: extrinsic system assay (EXTEM), intrinsic system assay (INTEM), fibrinogen assay (FIBTEM), and nonactivated coagulation assay (NATEM; ROTEM, Werfen, Barcelona, Spain; Figure 3).

### Assessment of Molecular and Cellular Immunological Biomarkers

Serum biomarkers will be assessed using the Luminex Bio-Plex Pro platform set for 27 human immune mediators (Bio-Rad Laboratories, California) for the simultaneous assessment of the following analytes: chemokines (CXCL8, CCL11, CCL3, CCL4, CCL2, CCL5, and CXCL10), proinflammatory cytokines (IL-1β, IL-6, TNF-α, IL-12p70, IFN-γ, IL-17A, and IL-15) and regulatory cytokines (IL-1Ra, IL-4, IL- 5, IL-9, IL-10, and IL-13), and cell growth factors (IL-2, IL-7, FGF-basic, PDGF, VEG, G-CSF, and GM-CSF). All procedures will follow the manufacturer’s recommendations, with levels obtained at days 0, 3, and 9.

The expression levels of immune mediators will be assessed by TaqMan quantitative RT-PCR (qRT-PCR) assays (Applied Biosystems, Foster City, CA) for each transcript, with retro-transcription set up with 1 ng/μL of RNA from PBMCs and from neutrophils. The reactions will be carried out on a QuantiStudio 3 Thermal Cycler (Applied Biosystems, CA) in standard mode [26]. Alternatively, qRT-PCR experiments for selected genes will be based on the SYBR Green (family of dyes for molecular biology) detection methodology using the SYBR Green PCR Master Mix and universal cycling conditions on the ABI Prism 7000 Sequence Detection System. Expression levels will be assessed on days 3 and 9.

Immunophenotypic/functional profiles of circulating leukocytes will be assessed upon short-term culture in vitro. The cell suspension will be stained with distinct panels of monoclonal antibodies comprising CD3, CD4, CD8, HLA-DR, CD25, CD19, CD5, CD27, CD38, PD-1, CD28, CD14, CD16, and CD56 for cell surface analysis, and IL-1β, IL-6, TNF-α, IFN-α, IL-5-PE, and IL-10 for evaluation of intracytoplasmic functional status. Cells will be run on a FORTESSA flow cytometer (BD Bioscience, CA). An average of 100,000 cells will be analyzed per sample. The phenotypic/functional parameters will be assessed using FlowJo software (BD, New Jersey). Leukocyte phenotyping will be obtained at days 0, 3, and 9.

The immunoglobulin profile will be assessed by multiplexed PCR using cryopreserved PBMCs [19,27]. Sequencing libraries will be produced and sequenced using the Illumina MiSeq 2x300 bp platform. For each sample, 1 million reads corresponding to IgG and IgA will be produced. The reads will be processed using pRESTO [28], Ig heavy chain genes will be annotated using the MIXCR platform [29], and clonotypes will be determined as described in [30]. The immunoglobulin profile will be assessed in samples obtained on day 3.

### Neutrophil Evaluation Experimental Design and Groups

Neutrophils will be obtained from healthy donors and from patients at two different times, T9/T18 and D9/D18. Neutrophils isolated from each donor will be divided into two aliquots and incubated in buffer (control group) or phorbol 12-myristate 13-acetate (PMA; activated group). Cells from each group will be submitted to the functional and molecular analyses described in the following sections.

### Neutrophil Isolation and In Vitro Activation

Cell isolation will be performed from peripheral venous blood by centrifugation in Percoll gradients, as previously described [27]. The remaining erythrocytes will be removed by hypotonic lysis. Samples containing >95% neutrophils and >98% viable neutrophils will be prepared in 50% autologous plasma and divided into aliquots for control or activation in PMA [28].

### Evaluation of Reactive Oxygen Species Production and Phagocytosis

The isolated neutrophils, after incubation in each condition, will be tested for reactive oxygen species production as described by Tahir et al [29].

### Evaluation of Neutrophil Extracellular Traps Production

At the final 20 minutes of incubation in each condition, an aliquot of neutrophils will be removed and incubated with SYTOX green (high-affinity nucleic acid stain; 10 µmol/L) and 4′,6-diamidino-2-phenylindole (blue-fluorescent DNA stain; 1 µg/mL). Fluorescence readings will be performed both by fluorescence microscopy and on a spectrophotometer [30].

### Real-time Migration Assay

Neutrophils from each condition will be applied to the upper chamber of a xCELLigence Real-Time Cell Analysis instrument and dynamically measured regarding the migration toward the chemoattractant N-Formylmethionyl-leucyl-phenylalanine (potent polymorphonuclear leukocyte chemotactic factor) [31].

### Neutrophil Proteomics

Neutrophils obtained in all experimental conditions will be subjected to cell lysis by ultrasound cavitation and then trypsin digested. Eluted peptides will be subjected to capillary chromatography in a two-column online vented system. The fractions will be eluted directly into the ionization chamber of an Orbitrap Elite mass spectrometer to be analyzed in data-dependent acquisition mode. The 20 most intense ions will be selected for high-energy collision dissociation fragmentation, and the fragments will be analyzed in the ion-trap detector. Mass spectra will be analyzed for protein quantification and identification using the Progenesis and Peaks software packages, as well as in-house developed R scripts for statistical analysis [32].

### Metabolomic Profiling

We will aim to analyze each serum sample collected from mild (T-branch group) and severe (D-branch group) case patients and healthy volunteers at each time point in the same manner (Figures 1 and 2). Untargeted metabolomics will be performed using a Bruker UPLC-PDA-QqTOF (ultra-performance liquid chromatography photodiode array quadrupole-quadrupole-time-of-flight) compact (UPLC-MS/MS). The obtained data will be used to build molecular networking using the Global Natural Products Social Molecular Networking platform, which gives a better overview of the metabolic profile and group compound classes in clusters. For statistical validation of the metabolite differences between the groups, the data will be processed in MZmine and analyzed by means of chemometric tools, as provided by the MetaboAnalyst platform. Based on this broad chemical outline, specific metabolites will be quantitatively analyzed using targeted metabolomics using a CLAE Quadrupole/Quadrupole. This approach will be used to validate and quantify specific components of interest, such as metabolites involved in the glycolytic and hexosamine pathways, and tricarboxylic acid cycle, among other possible biomarkers.

### Sample Size Calculation

The sample size calculation was based on the assumption that 20% of patients who are critically ill have elevated TNF-α levels compared to 5% of patients who are noncritically ill [8]. TNF levels will be normalized according to the study population. Based on a bilateral 95% significance level, a power of 80%, and a case-control ratio of 1, a sample size of 200 patients can be predicted.

### Statistical Analysis

Clinical and demographic data between groups of patients (severe vs nonsevere) as well as treatment profiles and differences in successive time points will be compared. To compare mean values of soluble biomarkers between groups, the *t* student test (or analysis of variance with post hoc test for multiple groups) or the nonparametric Mann-Whitney (or Kruskal-Wallis for multiple groups) test will be used as appropriate, according to the observed sample distribution features. The SAS 9.3 software (SAS Institute Inc) will be used with a significance level set at .05. The main binary outcomes will be analyzed by the estimation of relative risks using a log-binomial generalized model or by the area under the curve using trapezoidal methods. All personnel conducting laboratory procedures will be blinded regarding the patient status (severity of COVID-19). Receiver operating characteristic curves will determine cutoff points at which individual or combined scores of the molecular and cellular biomarkers become informative of the progression of COVID-19 cases, according to optimal sensitivity and specificity to identify the signature that best characterize each clinical status.

## Results

This protocol is in the data collection phase. Study recruitment started in June 2020. At the end of July 2020, 30 T-branch and 85 D-branch patients were enrolled.

We expected to complete all patient inclusion by June 2020, but with the continuous increase in COVID-19 cases in Brazil, the authors decided to continue including patients until February 2021.

Data analysis is scheduled to start after all inclusion data have been collected.

The coagulation study branch, with thromboelastometry, has already been completed. Preliminary results confirm deregulation in initiation of the hemostasis process. The results are currently being analyzed and prepared for publication.

## Discussion

This TARGET protocol was designed to standardize the evaluation of cellular and molecular immune biomarkers to evaluate their potential as prognosis tools during COVID-19 infection at different degrees of clinical severity. As the host immune response impacts COVID-19 progression, understanding the behavior of immune biomarkers at the different phases of the illness constitutes a crucial step in the identification of patients who will deteriorate and those who will benefit most from treatment. A simultaneous comprehensive assessment of the different aspects of the immune response was the approach adopted for this protocol.

An early study in China showed that the elevation of certain biomarkers such as D-dimer, together with inflammatory markers including ferritin, was associated with the worst outcomes after SARS-CoV-2 infection [33]. These measurements have been incorporated in some clinical practice centers, but their course during disease and actual value in prognosis are not yet fully described. Higher levels of a series of other interleukins were also described in severe forms of the disease [34], contributing to the concept of a cytokine storm, but their exact significance remains unclear. A thorough evaluation of different immune aspects could help to clarify their role.

Less information regarding the cellular immune response has been published, but its role is progressively gaining more attention from the scientific community, as some evidence notes that humoral immunity does not appear to be sufficiently developed after the first SARS-CoV-2 infection [35]. A simultaneous evaluation of these two kinds of immune responses in the TARGET study could help to understand this interface.

Thromboembolism is a recurrent finding among patients with COVID-19, which has made thromboprophylaxis a vital part of the available supportive treatment options. Unfortunately, there is considerable uncertainty about the degree of coagulation derangement, the possible association with the inflammatory response elicited including neutrophil extracellular traps [36], and the consequences of these alterations [13]. Further knowledge is needed to better understand COVID-19–associated coagulopathy to provide frontline clinicians with better treatment options to manage the ensuing clinical manifestations.

A metabolomics study of serum from patients with COVID-19 annotated 941 metabolites. The authors identified metabolic patterns in severe cases that could be used to propose diagnostic models capable of predicting progression to severe infection and assist in the treatment strategy [37]. Observed changes in the lipid profile (>100 lipids) cause liver damage, as reflected by abnormal serum levels of bilirubin and bile acids [37]. Another study revealed the consequences of treating patients with SARS-CoV with methylprednisolone. Disturbances in the serum lipid profile 12 years post infection were associated with long-term systemic damage caused by high doses of methylprednisolone [38]. Metabolomics is therefore a powerful tool for identifying and monitoring the prognosis and markers at different stages of a disease, particularly for unknown infections [24].

Similarly, neutrophil functional and molecular assays have been performed in patients with COVID-19 [39], clearly indicating the participation of these cells in the pathogeny, although no specific molecular model has yet been proposed [40]. Therefore, the proteomic approach coupled with further analysis at the transcription and functional level will provide evidence to correlate neutrophil activity to the disease severity and time course.

Only two treatments have shown substantial beneficial effects to date in randomized trials in COVID-19, with both exhibiting heterogeneity of treatment effect between subgroups. A preliminary report from Beigel et al [41] showed that remdesivir shortened the median recovery in a trial from 15 days in the placebo group to 11 days, but those on mechanical ventilation were less likely to benefit. On the other hand, dexamethasone demonstrated a reduction in mortality at 28 days, but mainly in those on mechanical ventilation [42]. One possible explanation for these findings is that an adequate immune response to stop viral replication is needed at the onset of the disease. The excess of inflammation presenting is detrimental. As the time when these treatments are initiated could be key, immune biomarkers that signal phases of disease to guide the timing of treatments could be extremely useful in clinical practice.

In summary, TARGET is a translational study that aims to verify the role of cellular and molecular immune biomarkers in the prognosis of COVID-19, opening up avenues for their use in clinical practice. The results that will be achieved are not meant to be restricted to the description of the disease pathophysiology, but to form a basis for future clinical studies that will ultimately help the clinicians on the front line.

## Abbreviations

ACE2: angiotensin converting enzyme-2
CNPq: Conselho Nacional de Desenvolvimento Científico e Tecnológico
D: days of hospitalization
EXTEM: extrinsic system assay
FIBTEM: fibrinogen assay
IFN-γ: interferon-gamma
INTEM: intrinsic system assay
MCP-1: monocyte chemoattractant protein-1
NATEM: nonactivated coagulation assay
PBMC: peripheral blood mononuclear cells
PMA: phorbol 12-myristate 13-acetate
qRT-PCR: quantitative reverse transcription polymerase chain reaction
RECOVERY: Randomised Evaluation of COVID-19 Therapy
RT-PCR: reverse transcription polymerase chain reaction
SARS-CoV: severe acute respiratory syndrome–related coronavirus
STROBE: Strengthening the Reporting of Observational Studies in Epidemiology
T: timeline
TNF-α: tumor necrosis factor-alpha
UnB: University of Brasília
UPLC-PDA-QqTOF: ultra-performance liquid chromatography photodiode array quadrupole-quadrupole-time-of-flight
WHO: World Health Organization
